# Supplementary material for: Plasmodium Condensin Core Subunits SMC2/SMC4 Mediate Atypical Mitosis and Are Essential for Parasite Proliferation and Transmission
Source: Cell Rep. 2020 Feb 11;30(6):1883–1897.e6. doi: 10.1016/j.celrep.2020.01.033 (PMC7016506; doi:10.1016/j.celrep.2020.01.033)
Supplement: Document S1. Figures S1–S5 [file mmc1.pdf]

Cell Reports, Volume *30*

Supplemental Information

Plasmodium Condensin Core Subunits SMC2/SMC4 Mediate Atypical Mitosis and Are Essential for Parasite Proliferation and Transmission

Rajan Pandey, Steven Abel, Matthew Boucher, Richard J. Wall, Mohammad Zeeshan, Edward Rea, Aline Freville, Xueqing Maggie Lu, Declan Brady, Emilie Daniel, Rebecca R. Stanway, Sally Wheatley, Gayani Batugedara, Thomas Hollin, Andrew R. Bottrill, Dinesh Gupta, Anthony A. Holder, Karine G. Le Roch, and Rita Tewari

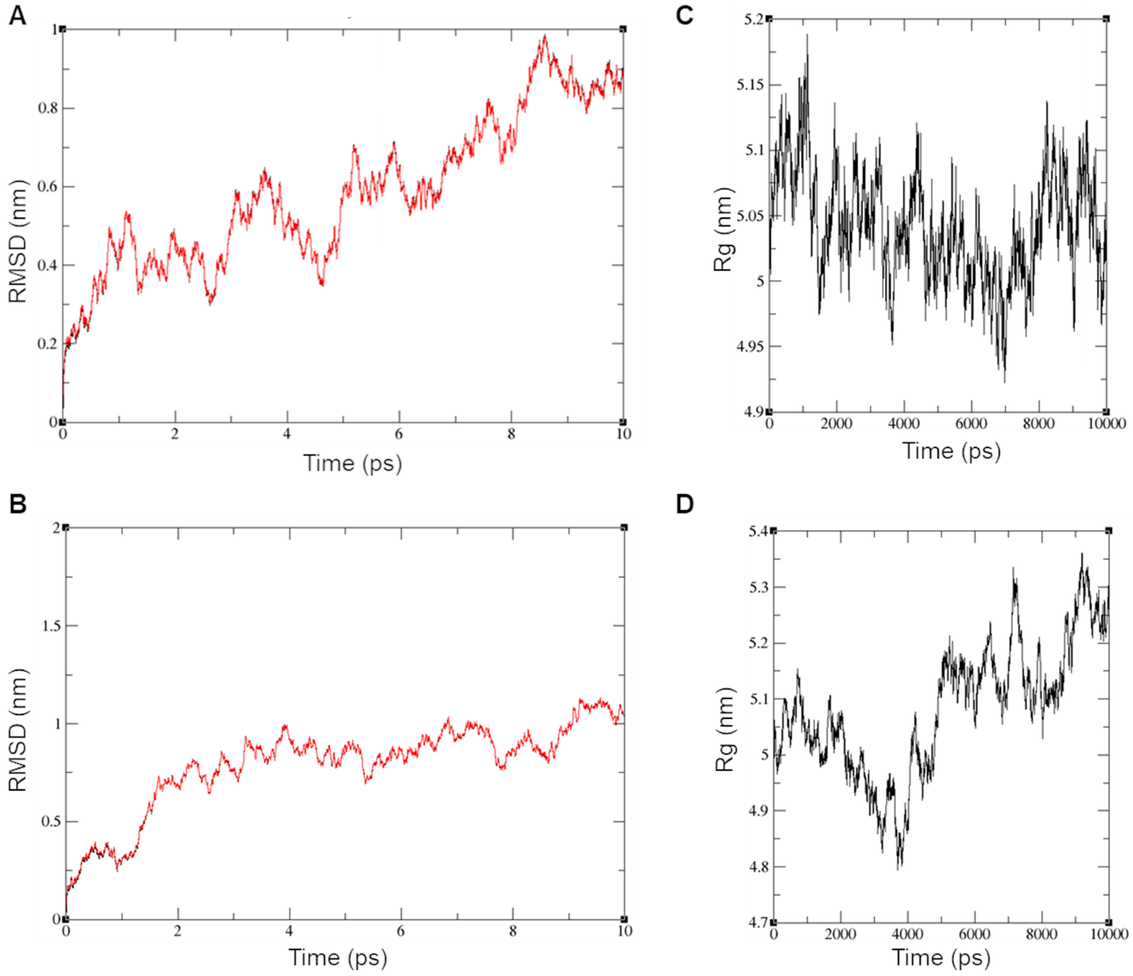

Figure S1: Related to Figure 1. Molecular dynamics simulation showing stable predicted 3D structure for SMC2 and SMC4. Root mean square deviation (RMSD) calculation using protein backbone structure, during the 10 ns production simulation for SMC2 (A) and SMC4 (B), respectively. Post 8 ns and 2 ns MD simulation, RMSD fluctuations becomes comparable and constant for SMC2 and SMC4 respectively. (C and D) Radius of gyration fluctuations within 2 Å suggest correct and stable protein fold for SMC2 and SMC4, respectively. Additionally, during a 10 ns molecular dynamics run the protein structure did not break, confirming stable predicted 3D structure.

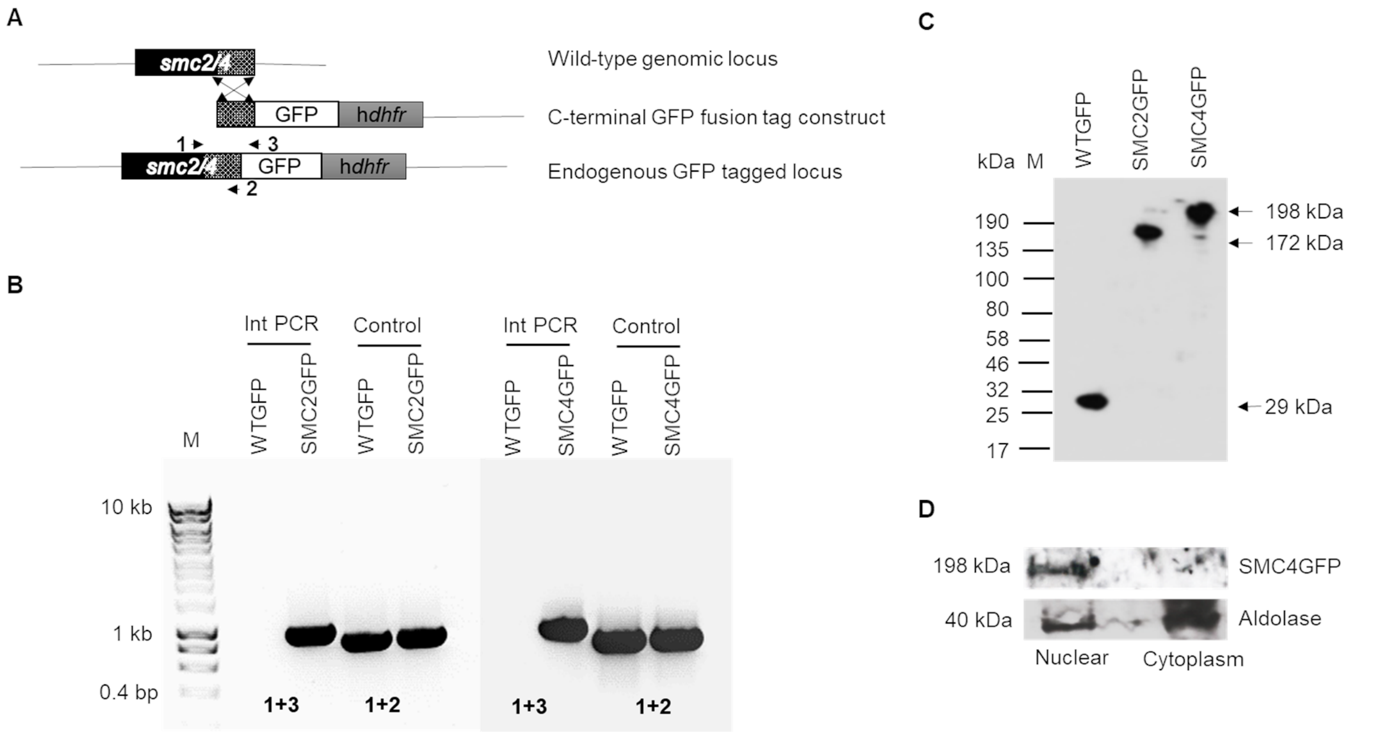

Figure S2: Related to Figure 2. Generation and genotype analysis of SMC2GFP and SMC4GFP parasite lines. (A) Schematic representation of the endogenous *smc(2/4)*, the GFP-tagging construct and the recombined *smc(2/4)* locus following single homologous recombination. Arrows 1, 2 and 3 indicate the position of PCR primers used to confirm successful integration of the construct. (B) Diagnostic PCR of SMC2GFP, SMC4GFP and WT parasites using primers IntT138 (SMC2, Arrow 1), IntT143 (SMC4, Arrow 1) and ol492 (Arrow 3). IntT138 and T1382 (SMC2, Arrow 2), IntT143 and T1432 (SMC4, Arrow 2) primers were used as control. Integration of the SMC tagging construct gives a band of 995 bp and 1006 bp for SMC2GFP and SMC4GFP parasite lines. (C) Western blot of SMC2GFP (172 kDa), SMC4GFP (198kDa) and WTGFP (29 kDa) protein to illustrate SMC2GFP and SMC4GFP in schizont stage extracts. (D) Western blot analysis of SMC4-GFP for subcellular localization from schizont stage extracts using anti-GFP (nuclear) and anti-aldolase (nuclear and cytosolic).

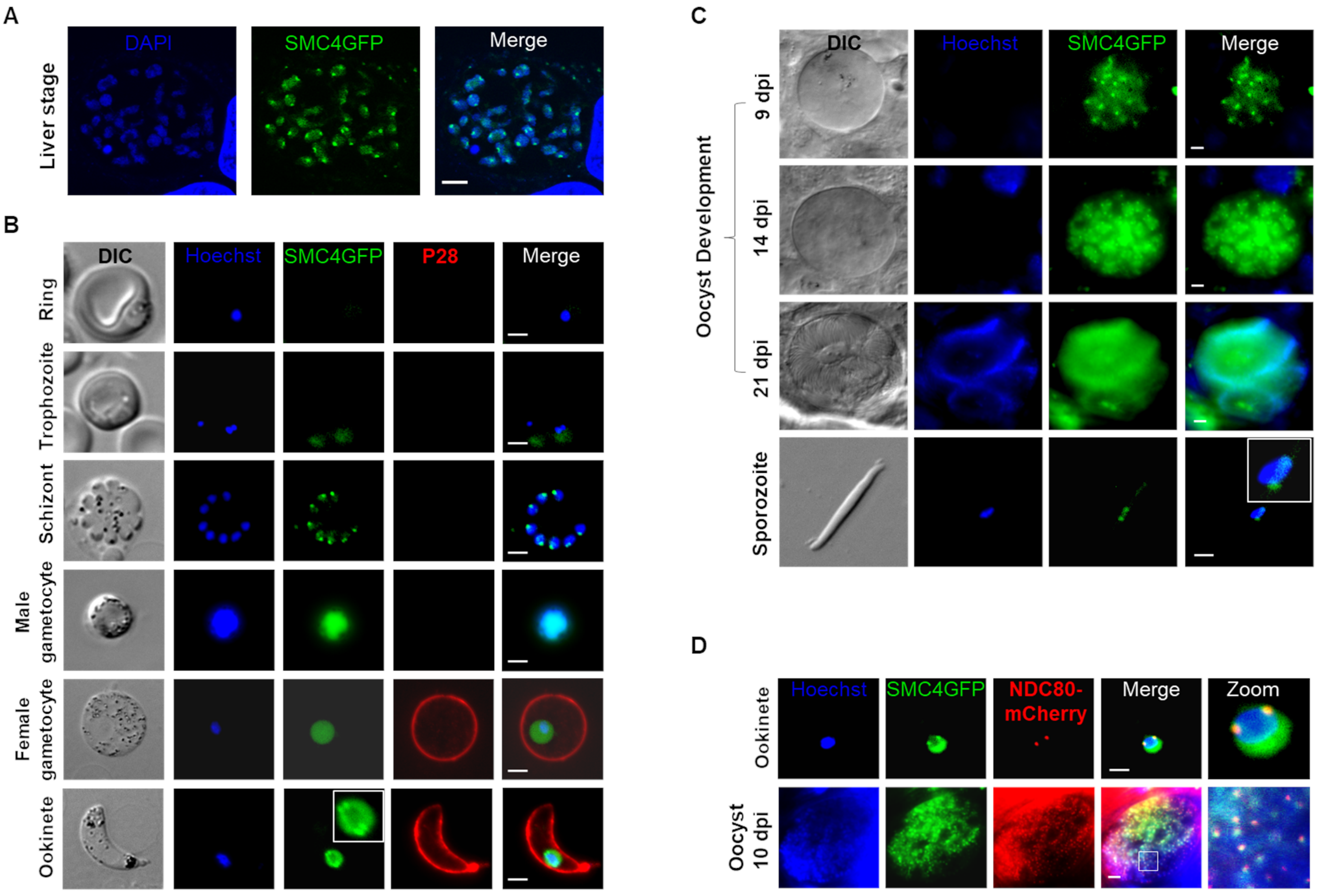

Figure S3: Related to Figure 2. Localisation of SMC4GFP throughout the *Plasmodium* life cycle as detected by live cell imaging. (A) Liver stage schizont at 60 hours post infection. Merge: DAPI (blue) and GFP (green). Scale bar = 2 μM. (B) Asexual blood stages and sexual stages at different time points. Merge: Hoechst (blue, DNA), GFP (green) and P28 (red, cell surface marker during female gamete activation, zygote and ookinete stages). 100X magnification. Scale bar = 2 μM. (C) Sporogony in the mosquito oocyst; 9 days post infection (dpi), 14 dpi, 21 dpi (Scale bar = 5 μM) and mature single sporozoite at 21 dpi (Scale bar = 2 μm). 63X magnification. Merge: Hoechst and GFP. (D) Live cell imaging of SMC4GFP and NDC80mCherry localization in ookinete (Scale bar = 2 μm) and oocyst 10 dpi (Scale bar = 5 μm). The white box represents magnified region. Merge: Hoechst (blue), GFP (green) and mCherry (red). 63X magnification.

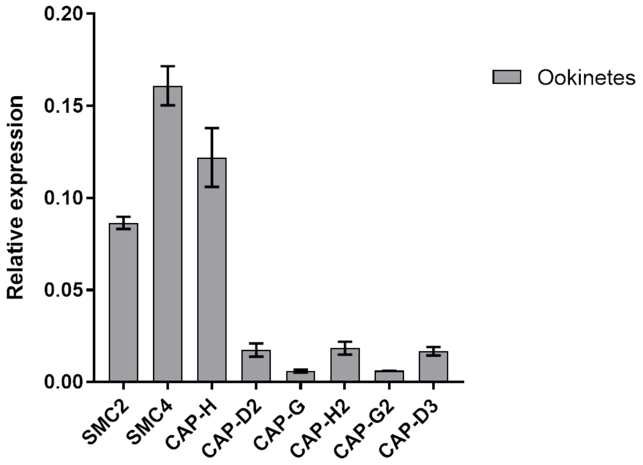

Figure S4. Related to Figure 4. qRT-PCR analysis of condensin complex subunit expression in ookinete stage of parasite life cycle. Error bar = ±SD, n= 3. Primers list has been provided in Supplementary Table S4.

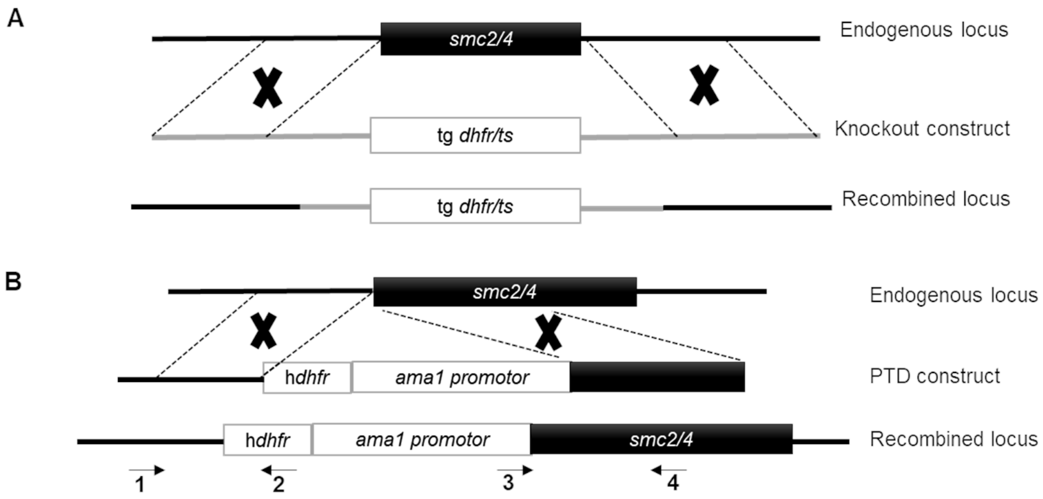

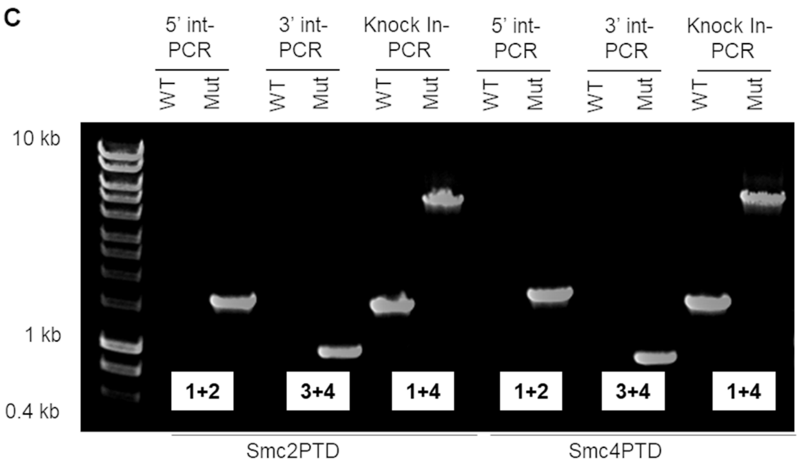

D

Experiment		Bite back (dpi)
1	WTGFP	4
	SMC2PTD cl 5	-
	SMC4PTD cl 3	-
2	WTGFP	4
	SMC2PTD cl 5	-
	SMC4PTD cl 3	-
3	WTGFP	4
	SMC2PTD cl 5	-
	SMC4PTD cl 3	-

Figure S5. Related to Figures 5 and 6. Generation of knockout and conditional knockdown of SMC2 and SMC4 genes using a *dhfr* drug-selectable marker or *ama1* promoter trap double homologous recombination (PTD), and genotype analysis. (A) Schematic representation of the endogenous *smc(2/4)* locus, the targeting gene deletion construct and the recombined *smc(2/4)* locus following double homologous recombination. (B) Schematic representation of the promoter trap strategy (SMC2PTD and SMC4PTD), placing *smc(2/4)* under the control of the blood stage *ama1* promoter by double homologous recombination. Arrows 1 and 2 indicate the primers position used to confirm 5' integration and arrows 3 and 4 indicate the primers used for 3' integration. Primers 1 and 4 were also used for Knock-In PCR. (C) Integration PCR of the promotor trap construct into the *smc(2/4)* locus. Primer 1 (5'-IntPTD18 [SMC2], 5'-IntPTD008 [SMC4]) with primer 2 (5'-IntPTD) were used to determine successful 5' integration of the selectable marker resulting in a band of 1518 and 1655 bp for SMC2PTD and SMC4PTD, respectively. Primer 3 (3'-intPTama1) and primer 4 (3'-IntPTD18 (SMC2) and 3'-IntPTD008 (SMC4)) were used to determine the successful 3' integration of *ama1* promoter resulting in a band of 1004 bp and 877 bp for SMC2PTD and SMC4PTD, respectively. Primer 1 (5'-IntPTD18 and 5'-IntPTD008) and primer 4 (3'-IntPTD18 and 3'-IntPTD008) were used to show complete knock-in of the construct with a band at 4698 bp (SMC2PTD) and 4653 bp (SMC4PTD), and the absence of a band at 1538 bp (for SMC2, endogenous) and 1493 bp (for SMC4, endogenous) resulting in complete knock-in of the construct. (D) Mosquito bite back analysis of WTGFP, SMC2PTD and SMC4PTD parasites. dpi = Days post infection after mosquito bite to mice.
