# Supplementary material for: Plasmodium Condensin Core Subunits SMC2/SMC4 Mediate Atypical Mitosis and Are Essential for Parasite Proliferation and Transmission
Source: Cell Rep. 2020 Feb 11;30(6):1883–1897.e6. doi: 10.1016/j.celrep.2020.01.033 (PMC7016506; doi:10.1016/j.celrep.2020.01.033)
Supplement: Data S1. Protein Sequences Used for Phylogenetic Analysis, Related to Figure 4 [file mmc6.pdf]

Data S1: Related to Figure 4. Protein sequences used for phylogenetic analysis. Protein sequences can be retrieved from EupathDB (https://eupathdb.org/eupathdb/) and NCBI (https://www.ncbi.nlm.nih.gov/) database.

>sp|G5EGE9|DPY26_CAEEL Condensin complex subunit dpy-26 (Kleisin Iγ) OS=Caenorhabditis elegans OX=6239 GN=dpy-26 PE=1 SV=1

MDVPSSSNVTGRRKRQVLDDDEDDGFRSTPLRKVRGTKKIRPADVVPETIMTKIGAHIDDIVNKKKVG
ELNCFEYKSPLEIHTIEDMIKAKASIQEMAVVLEGAQCIIGYRVDRLHHDVRQIDSALSSGTVMRDSNG
EEIHLTLESRKAKKKMAVVDGMNGMLDFLNNMDDALTTTELDADNDKNWKEDEENIAGEPRIDFKAN
SKDVDAFLQRDIFPEKLIYALSIKRATDLRADLLSDVSNYISADDTAHDLKDANIDWLRANPTFQKATKG
SVCNSSNSFHSLNYYGIHSPDGRTLMLHNRIADKNADDRFFTSDVSVSLVKNTRALLTNSLDKKPRIL
DNYLMLEVKDRPVIGRYKIMSKDVKKSTLPLAESSREKDLANLTFAEMNHRPSNLDMTVAGASDMSM
LPGNQGLPLAQGENDETIALDRLTPPLQSSVSQKASDEYVLPPLEANDLDEHLIGKLPIEPNEMDQTL
ANMFDKKLEVFNTSDTLESKVWKNGIRAEEWGEDDEAIMKNDTKHPRQAGIEGWIKATDAWTNYDV
VKMNVNREARSQLDENAIDEQESYRNMVPEIGKNLFLVKSDDYMNNYPGDRPADFTVNDEVSDVMK
MWSGEDSTAEDDVPLEQIQQEIREQVQQQDVDMEPIEEMDYDAGGAAFDVDFDDRLAAPVEVEEM
EGDNNRNDGRVADILFNEQMDETEVEERNEQDVQRELEDIALAADEVAELMTSAPPPQLVGPSAEM
REEIQNIGKNDNAHWVPPVVGDQERQAAVTAQRKRREKKAKSRKATVEDFVHYFRDIPDDEIEREITA
AKCSKIADEKSTFLSEQQLYLPTLGIENKPHVAFEMGLLGNSGMFFKKSYGKIRLERVKNQKAEQDLFI
DEARGNKDSDCLNWLLSFSGFRCMENPEPITGSDSDENLRTAVEQPFDDDFANDYYDEDRYDPNYE
QQLAAAQMGPDMQRKLALTASHINQMFPNIHSKRYGGEYGDSDDEFDDSFDRQSIQAKNLDAAKHK
KCLAEILKTDSLSMPSIQYVLEQLTSNQTLRMNNTTIRAADDRNETGRPATPTMEADKTLTSVFDYRS
PNKSNHDVNETMKALTEMPDYQAADERPNNQPTTSTYGTANTENRKVHINGCHTLLSLALSMPSRM
GETVRPSSIVSFLLHIANENNLQIVQDRSKRSWMSDFIVLNSSESLPRGLKMGRIEDQDEFWKRTQDP
DAIEGTASDANNVFSNLMRRPKAVPVRKGRGAGGQPTTSDLGAIVEEEEMEE

>sp|Q15003|CND2_HUMAN Condensin complex subunit 2 ((Kleisin Iγ)) OS=Homo sapiens OX=9606 GN=NCAPH PE=1 SV=3

MGPPGPALPATMNNSSSETRGHPHSASSPSERVFPMPLPRKAPLNIPGTPVLEDFPQNDDEKERLQ
RRRSRVFDLQFSTDSPRLLASPSSRSIDISATIPKFTNTQITEHYSTCIKLSTENKITTKNAFGLHLIDFM
SEILKQKDTEPTNFKVAAGTLDASTKIYAVRVDAVHADVYRVLGGLGKDAPSLEEVEGHVADGSATE
MGTTKKAVKPKKKHLHRTIEQNINNLNVSEADRKCEIDPMFQKTAASFDECSTAGVFLSTLHCQDYRS
ELLFPSDVQTLSTGEPLELPELGCVEMTDLKAPLQQCAEDRQICPSLAGFQFTQWDSETHNESVSAL
VDKFKKNDQVFDINAEVDESDCGDFPDGSLGDDFDANDEPDHTAVGDHEEFRSWKEPCQVQSCQE
EMISLGDGDIRTMCPLLSMKPGEYSYFSPRTMSMWAGPDHWRFRPRRKQDAPSQSENKKKSTKKD
FEIDFEDDIDFDVYFRKTKAATILTKSTLENQNWRATTLPTDFNYNVDTLVQLHLKPGTRLLKMAQGHR
VETEHYEEIEDYDYNNPNDTSNFCPGLQAADSDDEDLDDLFVGPVGNSDLSPYPCHPPKTAQQNGD
TPEAQGLDITTYGESNLVAEPQKVNKIEIHYAKTAKKMDMKKLKQSMWSLLTALSGKEADAEANHRE
AGKEAALAEVADEKMLSGLTKDLQRSLPPVMAQNLSIPLAFACLLHLANEKNLKLEGTEDLSDVLVRQ
GD

>sp|Q9Y7R3|CND2_SCHPO Condensin complex subunit 2 ((Kleisin Iγ)) OS=Schizosaccharomyces pombe (strain 972 / ATCC 24843) OX=284812 GN=cnd2 PE=1 SV=1

MKRASLGGHAPVSLPSLNDDALEKKRAKENSRKQRELRRSSALHSITPRRESLNNSSPFNSSHQVPV
LSNFEEWIKLATDNKINSTNTWNFALIDYFHDMSLLRDGEDINFQKASCTLDGCVKIYTSRIDSVATET
GKLLSGLANDSKVLQQTEEGEDAENDDEDLQKKKERKRAQRSVKTLVKDFESIRAKKFELECSFDPL
FKKMCADFDEDGAKGLLMNHLCVDQHGRIVFDSSDTVIKDLENKDVEAESQEAVVAAPIESHDTEMT
NVHDNISRETLNGIYKCYFTDIDQLTICPSLQGFEFDSKGNLDVSLLKSLSDEVNMITTTSLVDNTMEKT
DADAASLSSDSDGEEGHIVHALEEMAYDEENPYVDVVPKAMDESENPDFGVDTEVNMADGSTMNE
NYSIISTAAANGVYEYFDKSMKKNWAGPEHWRIQALRKNINNASTVFNSSNTAESSDNVSRSLSSTE
RKKRRELDNAIDFLQEVDVEALFTPATSSLKLPKSHWKRHNRCLLPDDYQYDSKRLLQLFLKPKMSVL
PNADGEGQLQLNKALDDENDLDGIQPHGFDSDGSDNVDEGIPPYGFGDSDSPKQTPLLTPPSSSGF
GDNLLLTARLAKPDMLNYAKRAKKVDVRVLKEKLWKCLDLENTIKENSINSHIEGSEMESEETNMPVK
SFFSTVNQLEETYEKKELKDISTSFAFICVLHLANEHNLELTSNEDFSDVFIRPGPNLTTLEALENDV

>sp|P38170|CND2_YEAST Condensin complex subunit 2 (Kleisin Iγ) OS=Saccharomyces cerevisiae (strain ATCC 204508 / S288c) OX=559292 GN=BRN1 PE=1 SV=3

MTTQLRYENNDDDERVEYNLFTNRSTMMANFEEWIKMATDNKINSRNSWNFALIDYFYDLDVLKDGE
NNINFQKASATLDGCIKIYSSRVDSVTTETGKLLSGLAQRKTNGASNGDDSNGGNGEGLGGDSDEAN
IEIDPLTGMPISNDPDVNNTRRRVYNRVLETTLVEFETIKMKELDQELIIDPLFKKALVDFDEGGAKSLLL
NTLNIDNTARVIFDASIKDTQNVGQGKLQRKEEELIERDSLVDDENEPSQSLISTRNDSTVNDSVISAP
SMEDEILSLGMDFIKFDQIAVCEISGSIEQLRNVVEDINQAKDFIENVNNRFDNFLTEEELQAAVPDNAE
DDSDGFDMGMQQELCYPDENHDNTSHDEQDDDNVNSTTGSIFEKDLMAYFDENLNRNWRGREHW
KVRNFKKANLVNKESDLLEETRTTIGDTTDKNTTDDKSMDTKKKHKQKKVLEIDFFKTDDSFEDKVFA
SKGRTKIDMPIKNRKNDTHYLLPDDFHFSTDRITRLFIKPGQKMSLFSHRKHTRGDVSSGLFEKSTVS
ANHSNNDIPTIADEHFWADNYERKEQEEKEKEQSKEVGDVVGGALDNPFEDDMDGVDFNQAFEGT
DDNEEASVKLDLQDDEDHKFPIRENKVTYSRVSKKVDVRRLKKNVWRSINNLIQEHDSRKNREQSSN
DSETHTEDESTKELKFSDIIQGISKMYSDDTLKDISTSFCFICLLHLANEHGLQITHTENYNDLIVNYEDL
ATTQAAS

>sp|Q8C156|CND2_MOUSE Condensin complex subunit 2 (Kleisin Iγ) OS=Mus musculus OX=10090 GN=Ncaph PE=1 SV=1

MRIPRSETMNSSFLKARGQQDVLSSPLERVPPASRPGKAPLGTPKTPVLEDFPQNDDEKERMQRRR
SRVFDLQFSTDSIHLASPNRNIDVSTTISKFTNTQITEHYSTCIKLSSENKITTKNAFGLHLIDFMSEILKQ
KDAEPTNFKVAAGTLDASTKIYAVRVDAVHADVYRVLGGLGKDTPPQGEESHSGDGSTLETERTKKP
AKPKKKQSCKTIEQNLSNINVSEADGKCAVDPMFQKTAASFDECSTTGVFLSTLHCQDYRSELLFPSD
MQTLSSGEPLELPDLGFVDMTDLEASLQQCVEDRPLCPSLAGFQFTKWDSETHNESVSALVDKFKK
NDQVFDINAEAEDDEEDVPDGPLVEDFVDNDEPDLSAAGDHEEFRSWKELCQVQSNQEEVISLEDR
DIQVMCSFLSMKPGEYSYFSPRTMKMWAGPDHWRFRPRPKQDATSCTEHKKKSAKKDFEINFDDDI
DFDAYFQKTKAATILTKSTLENQNWKATTLPTDFHYETDNLIQLHLKPGKRSLKMDQDQKAKTEHYEE
IEDYDYNNPNDTSNYCPGLQAADSDYEEADDLFADPVGTLDLESDPKTTQENGHISPENQGVDITTY
QELNLVAEPQKVNKIEIHYAKTAKKMDMKKLKQSMWSLLTKFSRKEADTEANHTESGQEGAPEEVAD
EKKLSGLTKDLQTRLPPLMAQNLSIPLAFACLLHLANEKNLKLEGTEDLSDVLVMQGD

>tr|P91663|P91663_DROME Condensin complex subunit 2 (Kleisin Iγ) OS=Drosophila melanogaster OX=7227 GN=barr PE=2 SV=1

MTLPRLETPLRRSAVGSYQEGVSRMLTPFNDDEAERREARRRTLLQQHHRSSTLESIEDNETIKNCL
ELYNGNKVSKDNAWNLMLIDSLANLLDHHHKRMSNFKMAGSSLEASSKVYGLRVDSIYLDAMRISAG
LSARTLTDKQINAAEDDDGPQGEQATGEGQDSAQQAAKEAAPKPKRQKKPISTVTKNRETLNSRLDT
APLQDPVFGKLNSTCGASINASNRLMHNILPSFDSELRLRTTYNFWNSEESTEEVQDHTTLNAEMEQ
WPATSLMSTNLMRKLLPHAERSNLRPLHTGYIITSAPNPKSANEKAAEVVQDEDHDEGLDNADDVCV
NKISMAFDINAECEPMPDLDGPPPLVLEVDSNELEELTAEEQMVINNCRRLRKQTEFIEDLRPVDGNS
KLEYSYRPMDQISQFWAGPSHWKFKRTRPRSTFSQTNGQVDTQPIRTQRAKKSAHLNANRRAKALD
YGNVTENFFQQLDTTIRQRKANFQKKWDPRKLILPTKFELDPDLFFKYESAPSIKLSKRAGEPDSDEG
GDLGIDMDADMHHDDDNDQELFNNEHFTDAVPANVSVIAAIAAEQAAEASMMNVSAGEIGLTQMNAT
CNNTVFEIGTEFEGAPSQVAKVIVPFAKRAKVIDMKNLKKSCNSLIQKQLLNAVPEETIPSHPKKKGEH
YSKGFASFQQVYQKLPDLLTTKMSDSLSPSVAFYAVLHLANDLKLRLIPQEDLEDFQIRQVLD

>sp|Q564K3|CND2_ARATH Condensin complex subunit 2 (Kleisin Iγ) OS=Arabidopsis thaliana OX=3702 GN=CAPH PE=1 SV=1

MDESLTPNPKQKPASTTTRIQAPTSPFFLGSNDDRLEREQARAARAAASRRRSVIFARGSQPETESD
PCFDKQQILELFQNCIKLASENKINQKNTWELNLIDHLCEIIKVEDENNTETNFQKASCTLEAGVKIYSM
RVDSVHSEAYKVLGGITRAGHDDGGDHEDAAGAVENATNQKKQPEKKISPLSTLEPSFDALNVKKFD
VAFAVDPLYHQTSAQFDEGGAKGLLLNNLGVYGGCQVLFDSQEIPGKLVSSANKHDKSETIDLSFVK
ECVEQMVLNMRKKDEIVPSLRAIINQFDEENQRPSDTFSCGQQTTESFDISHGNDASYADDDEGYEN
FGTSFDYEGQSGDVDENFGPNEAEPIYSNFHEEVEPASLQDMDSDDRLENVDDYLFLSLGISSKQNS
WAGPDHWKYRKTKGPDVQPASEIKSSPPAKKTRKKKQAEPELDFAKALEEEMPDIFAPPKNPKTLLL
PASRTPCQTKLPEDCHYQPENLIKLFLLPNVMCLGRRRRKNSGETSRQQPDDYEHGESWGNDNVY
DDDDGPFDDNENDQSDAEDTNTLISQPRQVNKIDVQYDKASKQVDVQVLKETLWECLQESHQPPIQ
DEEHQQEPPESRSFKVLLASFPDDCQAAERTQDISPHLCFICLLHLANEHNLSLIGSQNLDDLTIHLA

>sp|Q8BSP2|CNDH2_MOUSE Condensin-2 complex subunit H2 (Kleisin IIβ) OS=Mus musculus OX=10090 GN=Ncaph2 PE=1 SV=1

MEDVEVRFAHLLQPIRDLTKNWEVDVAAQLGEYLEELDQICISFDEGKTTMNFIEAALLIQGSACVYSK
KVEYLYSLVYQALDFISGKRRAKQLSLVQEDGSKKTVNSETPCETENEFLSLDDFPDSRANVDLKND
QASSELLIIPLLPMALVAPDEVEKNSSPLYSCQGDILASRKDFRMNTCMPNPRGCFMLDPVGMCPVE
PVVPVEPYPMSRSQKDPEDAEEQPMEVSRNGSPVPVPDISQEPDGPALSGGEEDAEDGAEPLEVAL
EPAEPRTSQQSAILPRRYMLRERQGAPEPASRLQETPDPWQSLDPFDSLESKVFQKGKPYSVPPGV
EEAPGQKRKRKGATKLQDFHKWYLDAYAEHPDGRRARRKGPTFADMEVLYWKHVKEQLETLQKLR
RRKINERWLPGAKQDLWPTEEDRLEESLEDLGVADDFLEPEEYVEEPAGVMPEEAADLDAEAMPES
LRYEELVRRNVELFIATSQKFIQETELSQRIRDWEDTIQPLLQEQEQHVPFDIHIYGDQLASRFPQLNE
WCPFSELVAGQPAFEVCRSMLASLQLANDYTVEITQQPGLEAAVDTMSLRLLTHQRAHTRFQTYAAP
SMAQP

>sp|Q6IBW4|CNDH2_HUMAN Condensin-2 complex subunit H2 (Kleisin IIβ) OS=Homo sapiens OX=9606 GN=NCAPH2 PE=1 SV=1

MEDVEARFAHLLQPIRDLTKNWEVDVAAQLGEYLEELDQICISFDEGKTTMNFIEAALLIQGSACVYSK
KVEYLYSLVYQALDFISGKRRAKQLSSVQEDRANGVASSGVPQEAENEFLSLDDFPDSRTNVDLKND
QTPSEVLIIPLLPMALVAPDEMEKNNNPLYSRQGEVLASRKDFRMNTCVPHPRGAFMLEPEGMSPME
PAGVSPMPGTQKDTGRTEEQPMEVSVCRSPVPALGFSQEPGPSPEGPMPLGGGEDEDAEEAVELP
EASAPKAALEPKESRSPQQSAALPRRYMLREREGAPEPASCVKETPDPWQSLDPFDSLESKPFKKG
RPYSVPPCVEEALGQKRKRKGAAKLQDFHQWYLAAYADHADSRRLRRKGPSFADMEVLYWTHVKE
QLETLRKLQRREVAEQWLRPAEEDHLEDSLEDLGAADDFLEPEEYMEPEGADPREAADLDAVPMSL
SYEELVRRNVELFIATSQKFVQETELSQRIRDWEDTVQPLLQEQEQHVPFDIHTYGDQLVSRFPQLNE
WCPFAELVAGQPAFEVCRSMLASLQLANDYTVEITQQPGLEMAVDTMSLRLLTHQRAHKRFQTYAA
PSMAQP

>sp|Q9LUR0|CNDH2_ARATH Condensin-2 complex subunit H2 (Kleisin IIβ) OS=Arabidopsis thaliana OX=3702 GN=CAPH2 PE=2 SV=1

MTSHGGGEVRGERIHTVQPERDLVANWEVDLSEKLEEYLLKICSGEITGNEEDGQIPVNFAEAALLLQ
GSVQVYSKKVEYLYNLVLRTLEFLSKQRDQEQSKGTSNENEASSSRQVDEEENDLFWNVDDIPVDT
KNRLDSSVGGDTCPSQFVKPPANLVVLEGDCLDTSGDGGELESYLLATTHLYRDFILLDPCDAVAVN
EFLGDNYGGKGRNSAHRGSSVRKSFHSSVGRSGGSARKSSVGKNQGTNVHLSPICGNGPNDQNC
DQGSQPPVFEDNDHGFDMDNEYGGAMDFSDTDADEDDPWKPLNPYEPGKLKVKPFKKVKILKKIG
WSITKDHMTSMFPLARPNGPISSELIEIWKMHGCASKDEQASQDIPYYEKLREMLVNGGNQPCGANG
NYNDNDKDNHDEANNGDFHDFGEHDGDDAEHPFMDEDVLNMNDGGAAEFHNYDGFENGESNCQ
ESLEDLCRSHLDALLANIAKSEKQTDLAARVSTWKQKIEQNLEEQELHPPFDIQEYGDRIINKLTVEES
GNVETFTDLMKDQEKHEVARAFSALLQLVNNGDVDLEKPGNSTNEPMCYTAVKPFSVRLLKVHNRK
NEKRGIHLPQKRAKSPITKGKSHESPPPKKRNTCSVSSQTRKVSLKISKINGVGVRCTPNSKKRRKGR
SDDVTEVTEVASIEKSLGKL

>sp|P34341|KLE2_CAEEL Condensin-2 complex subunit kle-2 (Kleisin IIβ) OS=Caenorhabditis elegans OX=6239 GN=kle-2 PE=1 SV=2

MTRNAPPGQESTDLAWLVTPAKDLVENFSIDVLKALAGYLEVIRQESEDTDNQVDAATTYRLFDFQR
ACRIIQGSCAVYGRKVDHVYELTISVVDLVENKGQDDGNTGSRRGAGRRKNFNLGSTNYDLADIDSL
KQEALANFEKTVKEEKKSIDAVRMVENAEVIESQYERKSCLVAKPTQFMFKLNYGQLNRTDEQILNAK
SRPDVIGKVKDFEIKKSKVKHDQQILYSHDCYRGNLDQFTLPGARWMPDNKELAANFGVADLEVELD
LEQEHEKISAYGPFKDPLSGREVVPPPRWFIEQEAVRQNQEIQSRATSRAITIAAKTLRDSQGFGSQP
TRLSQPFVERHRQSNHLNDFLSFVEGRVNKNRPSTHLTTGLVDMFVDNFGSVMQNDEPNTSRRPDE
NYAPMDFDDDFGGGGDDDDDDYIRNLSRRDEKRAPAPWDELDKNHIIWYTGDENLPVVSKPVKKITK
FQPKPAEMLARKQRREEKINKSRRDEFMETHDYLQDYYYWRSAARINPIKDWKIESLRTAILAEKKRR
IKEKTAKIREARIQNMQRKRTARVIPVEQFEPVTEDIPTSNRRTLGAEYDDVVDEDLAAEVELSMFGG
GFDDDEEDVRPRGERPPMAPNNLEFDALQTDFDIPPAEYVPLRFEDIDDAELNSVINLPGNLLIDKALP
LLKKFAENRTDREQMAYEMAKAYEDVDVAVSTLQEHVDKWHSRMEPILEEGETRKEYDVHAVGRAV
IGQYDIEGGTKRLLDLVMDRPWYEISRYFLSCLFMCNVGNVMVSEDMELPLEERINSMKITLLKRDMH
CEMFKEAGALDA

>tr|Q8INL2|Q8INL2_DROME Chromosome associated protein H2, isoform E (Kleisin IIβ) OS=Drosophila melanogaster OX=7227 GN=Cap-H2 PE=4 SV=2

MERILPEEAEAAYLSEAREQILEIAKNRPGTQVAKCIRAYDEQQDLASLVVLLEELSKNTDYSTDLRISL
GYYIEELLRHCLGRNDVSRRSAIVAAGSALQYCGKIYGDRVEYLCQVVEHQIEALLTSELQKETPSGS
AAPEKNERRPEETRKRHPKKLTNKEVDPYLLTLEPKRFKTMSDDKRFNAAGFVKCTRNRTIEYLYQD
HTPPNLWKHAPIVDPHNPYDQDEKKQYKMFTYHVEHRYNTLLPDIPFERLNLIKEYVHTNQVNTTEIL
NEHMTTKEYLDEYIALENQMLAARYGAIVTRRRRLVDSARFMEDNLDEGLAKKMCMDKNLPMDTNE
TVLIDQSLVDENSRSLTTAESTMGISQAENSTLKSSEVEATLSVSHIENSLSDSQEENPPLSSTLAINES
SVLDSTRVDPIKDLTLDELLLIDSGISMEELSDIQMHTAGQSFDDEGVVLSDLEDQRQLSPMLQMVSP
SMEAKTLNIIEMDADLIMNVPREVSYPILLNVMGLPIKRLRRKCIFKLPPEFDLFRQARLPIKREGQQKS
PTTPRTVLQIGREAPQNEREPGSPCSLEFDEDLNFLGFRQRRPTFDSGFDIEEPRVSSCVSTIGEVKT
ELEDEIEANTNNLTDASASDALNENVNDTKESGLGDSLAQELNASTEPNAENNTANSSQLEVTATESS
GTLSGLETTVDLGMESALVSSVLDLSTEPSAMDSNIINVTQPDNSDLEISTVQDQDTLHDEADANDLS
AETPNPIADSAVDDCSLIRDWHRRLAPALEAAHERQNFNIKDLGTEILDICKAGNRTATLADVMADKDP
SIMCRYMLASLVLTNHGNVSLDFENRDKSKPIDMSQFRMHLKSMKRMEINPEDDVGNINAAQSKSTP
RSKQLNDSAHKPRTSTTASAPTKRKSAENSLSEVFAKTVRLIQPIPKMWPTPSDADSGISSMGSSLAS
TARLK

>PF3D7_1304000 | Plasmodium falciparum 3D7 | condensin complex subunit 2, putative (Kleisin Iγ) | protein | length=1024

MKKLGVNNAGENKGNSIQNKDGTNKKTIEVNKNMRRLTFLNNESNDSIEGNKNKYDKNKVKEINDVF
KNCMVALSHNKICTRNAFDIHIIEHLEDLINLNDEEIPEELNDEMIENGEFNLSFTRASKAIEGATKVYGY
RVEAIYDQTYNFLTNMNLAKQFELDNDMMDDNKNTIDPLNKRMRKRKLTYLQESSTLAKSSDITVDSL
SLSNISVDTFFLKLNSTYDHSCGQKYLLPNLNLNNDLSIQFDGDIDVCEYKKRRKTLDDIGKDGVKDKE
DGIHIIMKNKNDDNCDTYDKIICNNNDINKVDEKKNNDVDICENRNKKNESCNIYVDTFNVNEHKEFFDI
YKKKLFLNADILKEILYGGPDDFNSLHICPELDYFKEELKKHKLKRTDSKDTDDVDKEDGDGNLYNDD
DIGNTDKNNIINFYDENLGCNMSNNMLMESIYNSDALNTDRKNIFPENLGMSCGNLNEEKKNNNMNS
SNNNLNNCNYDNNDLYLGDYRMDDLNIENVMQESLGFDNLNYSCDKNMNFSNNNGMLLQQSINMM
DGMNFPELIKSENKELDLGNTQINNEHYLLKNNYDIHKRHSIISEVPDDDTLWNRSVMTFENRINAIDIN
NELNYHYYMPNKLMMMGNFRNLIDINKNVTNNINNNNKSTILQNIIMQKKMKYAFDVTSIDFENLYTEN
NNIELSTYDLWKKEKKKYVSNALFCIDQTSYIFDTRENCVNCVNTVTDKVMKFSKFCVYPDFLHACNK
NRNKHNNNNINKNHMNVILNEVNNDNILNDIDPINQDDFNNNIDNFDMENMDDMNDGHLHEGLNEAI
DKYYNMDFDNIWANNDNKNNDNINNNNNLNNSTYNNMNSHNNNNNNNNNNLSLFKKHSNFFNTALF
QFNHSNTFGNVPFENVSKFVDVAKIKKILCDIVKPNEEQKSNTNDSLCEYQTEKDNQIVTYTAEKTTTF
EEIVKETTAKLNESEASSTSIHMLFVCLLYTCNDQELLLEKIPNQNNFYVRYGLPVECHVNHDDIPMLK
N

>PBANKA_1402500 | Plasmodium berghei ANKA | condensin complex subunit 2, putative (Kleisin Iγ) | protein | length=943

MKKLGVSNKTNTNFTQIKGPDPIFKNPIEFNKNLRRLSFLNNKDEDLNKSEKNKVKEINDVFKNCMAAL
SHNKICTRNAFDIRIIDHLEDLVNLNDEEINEELNDELLETGDFNLSFTRASKAIEGATKVYGYRVEAIYD
QTYNFISNMNIAKKSETNDDVIDEKKHANEITNKKVKKRKLEFFQESSTLAKPSDITIESVSVSNISVDTF
FLKLNITYDHSSGISYLLPNLTLNNDLSIQFDGDIDTCEYKKKMKFEEKLGKQNIERRNEKNCGSNDEL
NDMLQKNESPVKVGEYVTFNDNDKIMAREYKSKLYTNSDILREMFFGNEIEEFNNLNICPELDYFKEEI
KNLKLKRSDSKILDDEDIDNYNDDDTGLDKFSKKKNELNLDGNMSMDNLLGDMDGNNNDDGNNEHM
NDNANYGDNNKILESSFNNNLNFDDCNIDDLNIENVMQESMVFDNMNLNDSLNNNLNLSQNILSLHH
NSNILGSSIPLPELMKSENKDFSLINSFTGNFNFSSQNMLFKNQDKGSPSKPKNMLSIIPDDDTLWNR
QAISFENRLNAIDVNSKFNYHYYNPSKLMINGNFANLMSMAKVAFKNKQGPLNALTNKKLKTSFDITYI
NFENLYIEVNDVELSAYDLWNKEKKKYISNSLFAIDQTSYIFETKDNCINCVNTVIDRIMKFARSPFIESQ
NFNTDIKTNVILNEINNDYIGNDLDQINNFERKQSENMFMDNMDDGQDYQMHEGLNDSIDKFYNMDF
EDIWQNENKNDITKFGSKNDNTSIFQLHQSMGHTNSLGSVIAPDNLPKFVDVSKIKKILFNIVKPDENE
ENVENGKSEENGKSEENGKSEENEENKKNSDSSKQIVPYEGEKTTTFKSIINKTKTKLTESEVNGTSI
HMLFVCLLYTCNDQELLLEKIENEEDFYVHYGLPVEFHIKQDDTRMLKN

>PY01534 | Plasmodium yoelii yoelii 17XNL | CCAAT-box DNA binding protein subunit B (Kleisin Iγ) | protein | length=886

MKKLGVSNKANANFTQIKGPDPIFKNPIEFNKNLRRLSFLNNKDEDLNKSEKNKVKEINDVFKNCMAA
LSHNKICTRNAFDIRIIDHLEDLVNLNDEEINEELNDELLETGDFNLSFTRASKAIEGATKVYGYRVEAIY
DQTYNFISNMNIAKKSDTNDDVIDEKKHVNEITNKKMKKRKLEFFQESSTLAKPSDITIESVSVSNISVD
TFFLKLNITYDHSAGISYLLPNLTLNNDLSIQFDGDIDTCEYKKKMKFEEKLGKQNIERRNEKNLGSNDE

LNDMLQKNESPVKVGEYVTFNDNDKIMAREYKSKLYTNSDILREMFCGNEIEEFNNLNICPELDYFKE
EIKNLKLKRSDSKTLDDEDIDNYNEDDDTGLDKFSKKKNELNLDENMSMDNLLGDMDGNNNDDCNN
ENMNDNGNYGDNNKILESSFNNNLNFDDCNIDDLNIENVMQESMAFDNMNLNDSLNNNLNLSQNML
SLHHNSNILGGNIPLPELMKSENKDFSLINSFTGNFNFSSQNMLFKNQDKGSPSKPKHMLSIIPDDDTL
WNRQAISFESRLNAIDVNSKFNYHYYNPSKLMINGNFSNLMSMAKAAFKNKQGPLNALANKKLKTSF
DITDINFENLYIEVNDVELSVYDLWNKEKKKYISNSLFAIDQTSYIFETKDNCINCVNTVIDRIMKFARSP
FIESQNFNTDIKTNVILNEINNDYIGNDLDQINNFERRQTENMFMDNMDDGQDYQMHEGLNDSIDKFY
NMNFEDIWQNENKNDITKFGSKNDNTSIFQLHQSIGHANSLGSVVAPDNLPKFVDVSKIKKILFNIVKP
DENEENAENENEENKKNSDSSKQIVPYEGEKTTTFKNIINKDANFVNRAHELYICILYIYFLT

>PVX_122040 | Plasmodium vivax Sal-1 | hypothetical protein, conserved (Kleisin Iγ) | protein | length=928

MKKLGVNTKAGANLTRIKENENLFKKPIEFNKNLRRLSFLNNKNNDANQKDHQDKCDKTKVKEINDVF
KNCMVALSHNKICTRNAFDIRIIDHLEDLVNLNDEEINEELNDEMLETGDFNLSFTRASKAIEGATKVYG
YRVEAIYDQTYNFLSNMNIAKQSEVNEELVEEKKNANEISNRKIKKRKLEFLQESSTLAKSSDITMDSV
TVSNISVDTFFLKLNSTYDHSSSNSYLLPNLILNNDLSIQFDGDIDACEYKKRKKMEEATEEGVDEDTA
ATQTSGKFPPMIRSSSSYDNDCSDAMVKCYKKKLFLHSDVLRDILFVAGNEDFNSLNICPELDYFKAEI
SKHKMKRSESKELEDGEQGNDEDDDDDDEEDDRRYAYNLNEEEHMSMHKGDDYGGERAKNNLAS
SVGGNLTMDNLLDDCDFHNASVGDGRMLNSSVNNNSMHFNDYKIEDLNIENVMQESLAFDNMNLND
SVGNNLNYSQSILSFQQGSNTMTGLPLPELMKSENKDFSLMNSLGGNFPLSTQNSLFFKNQGGSPT
RKGLISTIPDEDTLWNRNVMTFENRLNAIDVNNKFNYHYYVPSKLMAHGNFRNLMDIAKGTHKNKHM
VLQSVVQKKVKASFDVSLIDFENLYKEINDVELSTYDLWKKEKKKYVSNALFSIDQTSYIFETKDNCINC
VNTVTDRIMKFSKAPMIAPGDYCGDLKLNVVLNEVNSDFVTNDMGQIHYGEGRNMESTFPENMDEH
QDCHMQEGLNDAIDKFYNMDFDDIWQNENEHNLSKQGSKNENASALQLRQRVSHGTTLGNANMEN
VAKFVDVSKIKKILCDIVKPAGKEATGSEGPAQRNDQIVPYVEEKTTTFKDIIIATKSKMNPEEVNGTSIH
MLFVCLLYTCNDQELLLEKIPNEDNFYVRYGLPVEYHVKPDDMLMLEN

>TGME49_288930 | Toxoplasma gondii ME49 | hypothetical protein (Kleisin Iγ) | protein | length=1185

MVARATDGGDTGAPAGSETHPVVRLGFRPDGKRRLSVVSGPASLSTGDGAPSGESRRGVGGTTGA
SLAGHAPGALARQASSLNSASSSFSSFASSSAFPASLPFSSSANRPGFARPTARAASGAGRALAGRL
LVRAFFDDMKTVSMQRINQKNAFQVDLIDRLALVVHQQLVKTDNAVLTDGTDGQAPALLDGASSPDL
SSTSELSRTGRGEEGEEGITFTHVSSAVEGATRVYGYRVEAVYDQTYHVLNLMSSSRQGQGGEDAG
DGDAAAHPTRRGRHHQQLALFKKGGASTLAPASEITESQIEKDSCVDPYFLKISGMFDQAGAKGLLLA
NLEVDTSLRMKLDGECRAFPTGVLRNGREKGNAAREETEANHAPEETAEVAEPSVDCAFLRDLLLAG
ETPASVLALDICEKPIGHFRELLQSLRRGRDVKAGDALSEAEEETNELDVDDDMLDVDMQGTTLGDS
GFQQATQEDEELLKIGESYMDDEAASGGLREVLEAQEAESREVTQDDELFGDGVDFFCDGHVGDAD
DDRDGSWDRPRGEIRERDAEGERILYEVAADESSASSCTHPKSYSFDRLVPAFSQMLGVPAGGHGA
QLAAKRAFGLVPTAAGSQGGGDPEGRETGRRRGSGEETSSSGCVDFFPLSAKGSAAAVGRPSAPS
ARLTVKERQLRKQEQFREMLDPFRVDMSNLDTKSGGLPLGSKFQCYVPKPQENVTVASDFTSSPFL
HSPHLLVSLAMVPSKEIRLLRHTGPSVGDGWAPDGAGNSGGRAGRDLGRGDSQAPDGPWEATFID
AGDDGGLEDDSFWSAAAHGPLLKESALEAADAKAQTLLEGGGGLDRDWSDTAWAGGENDQASSYI
AHLFERGDAASRGGAAGLSLGDIVLLAEPQKVGVSELRLSIPSRYVDVAVVKKALKTALGVVPDFSAD
QDAENSALRRKRKRDMLTAPGEEEPQEEDDVDSCLDSVDGETKEPCRDLALWNPRGRRDGIDFETL
CAETTRKLPSTEKANLSPQMLFVCLLYTCNEETLHLEQSADFSSFSAFVHAPTDWHMRDDAEAVRVL
DTVRHYTPLALPAVPEKKKKETLSLEAPSAESAADDGGAAGDADDAQSPAKRRGALGDEERRQKRR
REGRGGKHEEGNRKGHRDEETGSKKRRGKKRRKEDSDESSDGSDDEEAAASEDRDGSC

>cgd3_3960 | Cryptosporidium parvum Iowa II | Condensin complex subunit 2 (Kleisin Iγ) | protein | length=901

MARLSLHPRIFESEYSAETDNYLEENVSNLNKNGWSGRKIQKSVLPNKRRSISIGQSRNDDSETTRLS
RTSINLKGQIDQNKSFFSKSSSDQLPNEELDKLCNQCLNLLRQNKISSKNAFDILLIDHLNDIVNVQDSQ
NEEEKSNIIKKENKENVSKINNNSKEKNIEIDKSKMNKGEFHNEMISSETTSQDTNFQKFQRAAVTLEA
SARIYGYRVDSTFDNAYRILSNIKSGQILARNDSDCEEDEKDQQEDDSSGNRLEESSKKKKYKRNLIF
SGENNTIVSNPESITLKEFEKTKEMSEKPFFVNFLNDKVKIDNIRFGLDYNNIGSISSMLMNNLELKDSL
YNRDKSASLTFNISGRNNLINSNNFSCSTICKDELFKVNKKTLDLLLPNPNENFGKDISNIVCPNLSRIF
DSIQEITENYGDIKQVKDSTSIEEYQLNEQVIRRFFEFTDPSFENDSLESENNFLKKHSQSMDDIEYVN
ENQDLEFLNNFEEEEKVELNMGLQDFQMHVERVIKDLNKTESSEHILEDLDQNKDIEIEKKSGEFKFS

KIGDVLDFLAKKSTKSLEFFNERKKGEKSNSVFGQKLRSKSENTNFEIPSPLLTNNIFNGIEWMNNLPK
IKQTQINSSRSKTKNLSSNKLFVGKNSTTSIFNYNLNHLFCLANLTGVKINLRSIHKAENSEETAGQNYI
TNDILIKTNNSQHINKNILNNSMSEQTDLMVNHCEIEDLMQVESYLQPLSQEEFSIDNGKDILIQSKLLLP
FDEDKISDTDILLEKSSKSLHVRDEYNSSLKFSKSSNHVDIDLIKNVLKNSIQILQSDDSNSLTLFNIINQS
RKLLQQDGLNSVSTGIFFICILHICNENNYSLNLSDQNANSDSPIELNSENYVHIMLNLNSSVDLQK

>ETH_00039510 | Eimeria tenella strain Houghton | Ccaat-box DNA binding protein subunit B, related, related (Kleisin Iγ) | protein | length=122

GFDEEEPLEAAAAAAAERVSFCHLSSAIEGATKVYGYRVEAVYDQTYHLLNGLSSLKNSSNGVEDDE
EQQQQQQQQGSKRKQRQQQQQLALFFKGGSSTLADPQDILNEQQDTGVLIDPFFV

>BBOV_I002180 | Babesia bovis T2Bo | hypothetical protein (Kleisin Iγ) | protein | length=726

MASRKRSRDVDSSEISEAPQPVSKKVTGPSGNTQDLLTLFTDCMSALSTNKICSRNAFEVGIIDHMTD
LVHLDDGSVDDDVVELLPEDTSSGASRRLNFTRASKVVESASKIYGYRIEAIYDQTFNVLMSMNSANQ
ADGTSGSTSATKPRGRHRVKIDLTTSSRTLAPESEVTLTEIPMDNVILDPYFLKISSMFDHSGAMGLLLI
NLQVTDDLSLDLDGDSLVFPPPRARSEHDDSNVILSRDAVKKCFFGNTEPKRMEILPEAAYFRQELER
LREMRRRKECGDVIASDSDGEINPEPESKHIDFFKVRQPIAPIEDSLEPMDAMGHIDDVPEDTVHDMPI
SGTPPDTGSDTGSVPNSTGIALRQRLAEIDLIGGSQFSYYTSVPVKTHVPSSKNKGSDTEPDDTNLKT
QRPVKSRQNITNDLESYLRNIDLEEKIASVELSTVFTTPKAVGKKAPSTFAASDFTGAIYRFNDTFLTRL
GLLGNRCLRFVSDHEWRNGPNTDSSVPILQVHYCADIDAPYSTMRISENFSWRLDEQVGLLDEDDM
WNASNDFEDPELPLTQDGPVVANIESMYESEPGEPLLALSQHADPAAISQWPREGAIVPAPVAAYVDI
FKIKKTLCGVIVPPPAFHEALEDKQQIDKEHGSGNTQSEYSCGFQDAVTDTVSRLMDSDVAALTSHIL
FVCLLHVCNEQDLLLKQSRPLEDFVICAGAPKEQHLGDVQGSL

>PBANKA_030180| Plasmodium berghei ANKA | condensin-2 complex subunit H2, putative (Kleisin IIβ) | protein | length=775

MSTQEDEVRLLIQNLQKCNNTNECINFDLASTIQEFLNSLDKNSFEDIDKTIRENEKDKDLMNSFTSAAI
FLENCVKILGLKIEHLHNLAHNTLYNIYKENKNSNSNKKQLLIIDEEEYLYINEIKNLKNTITENDIIEEDLL
VKTIPLPTFLFTDHIRVKNKTENHKNIIKYDEKDNFLINKKKLLNEPDIENIPYIDTLGNESINSIETKIMDLN
STNSYKNMDNLNLIDNKQTIEISSVNSLNFDKLFLENDGIILLDINDYNIFINDEYDFTLQNKNSTILFEKY
EFFSRNSIYLSNNLTEYIHEQNTIQHTYKINNIYDITSLRLCTDTLLFKTDFYSYDIALDIIKNKNYLINKFE
RQKKKLYILDETIHKDHKYNTIYKQNADYCDYCGSIITSIIEPNEPNNRCIYCNNNTQIDRNEKNGLYKKL
PGYYNLSCYNITETEDFLTYMQPNKIIDIIMKNEINDINLDSNSTTNKFEENIIDPIFHQNNDFTDKGDNY
RLSNDQKLFRQIKIPSLYIQKLGLNIDYYYLEPLIYNLIKNLKKEKNVDRFFSINFYDHNENYDIEILKDDY
YHEIKDEQNKTIQETLNMDTFINIKSIDNHVKNFPTSILKKTDSNTSLVFSFEDKIQDRVNAWSNFLEEK
LVILKRQPQYNVEYYKKKILKYIINNGDNIYFPDLIKNDEKYQIYRNFLTTLMLINTNKLNISEIDQQKHSN
NITNYQINIKNINVNEYMNISSAFDNTKFTINDKKRKITEKSDNIDNSFHLEKKNHI

>PFB0185w | Plasmodium falciparum 3D7 | condensin-2 complex subunit H2, putative (Kleisin IIβ) | protein | length=797

MSTTDELNLLIQNLQKCNNTNECINFDLSSTIQGFLNCLDRNVLENIDKGLGENEYEKEVVDNFTSAAI
FVENCVKIFSQKIEHLHNLAHNTLYNIYKENKHNSSSKKNQLIMSDEEEYLYINEIKNMKNTQHDNDIIE
DDILIKTIPFPTFLFSDNIKKTKDINEDKRKTNFNNNEEDKEKDNKNKDNDIDAINEFEITDNNSVNTLNF
EKIFIENDGILLLDINDYNVFIDDPYNFSIQNKNSTILFEKYDFFSRRSTYLSSNTLSKYVVENKNMDHIY
KLYNHITDIINKNICFDIFLFKQDFFDYDFSLGILKNKKSILNKFKQQQKKLHPLEENTHMDTHHINNNHH
LQKYDLNRPLPNYYMLHCYNIKNYQDFFRYMQPNYILEIMKRHIIKEIYNTNQQERAIQKEAYEIYNEQT
KKKNDHKENNNIDVPKYKDNTKCYDSPFYNYYISNNIIQFDHLIDDDMIYFDEYFYKSLILYNTNINDLHK
NTNNNQTNDETNIINNMKDEKQKNLIIYSNINNFSNDQKLFNQIKIPELYIQKLGLNFSYYHLEPLIYNFIK
TLKKKNDFEKFFSVNLFDDKPIYEFDILRDDEYDEQKNEDNKNHIEENINFENITDKNILNDEMDNIPIAI
FENDHLDNTFIMNDDQELQDRVSKWNAFLEEKLEILKRQPKYDLDLYKKNIINYTINNGENILFTKLIKN
KDKFEISRNFLTTLMLINADILNIKKINKHKKSNNISNYEIHIKKENLQQYLSISKQVQNKSFLIKEKKRKK
NKQHLTNGMKDTSKKKQKI

>PVX_003630| Plasmodium vivax Sal-1 | hypothetical protein (Kleisin IIβ) | protein | length=784

MSTPDELTALIQNLQKCNNTSESINFDLASTIQEFLNCLDRNVLEELEGGAHEGEKERESDREREKEG
EREREQDVANSFTSAAIFVENCVKIFGLKIEHLHNLAHNTLYNIYRENKHSNAGKKHMVMSDEEEYLFI

NEVKNLKSCPGESEPCMEEDLLVKTIPLPTFLFSENVKKKEGAAEVDSELGIDLSADLDTPRRSDNGS
GTDDRRGHSNLSKESEEAAREANGETDSIASSPAWRKGENDRLTEEAEEVLEQNSLKPLNFDKMYL
ENDGILLLDINDYNVFINDEYDMSILNQNSSMLFEKYDFSCSRHSTYLSPANLTKYIEREKTVGDIYRTY
HFNDILSDDLCSDVFLFKSDFSHYDLALGIIKSKKYLLSRFKEQKKYLYVLDENAHMDKDRVGTSTIEK
NDIKRKLPHYYMLNCQNIKRAQDFFTYMQPSQIIDIITRNARRRYLSCLPSGETSQGGQSGNAAGADS
PVGGAASHTSPPATPAPPPPGRSTDQKLFEQIKIPDIYVQKLGLNFRYYHLEPLIYNLIKELKKKKNVEK
YFSLNLFDEQNNYDFDILQDEEYADEQNEGNAAAEGEGNLTDENFLDVRSLNGDMVGGMMGGMMG
GMMDDIPLDVFEKKDSSDAFFASFDEDIHDRVNKWNAFLEEKLQLLRSHPKYDVDQYKKNIIHHTLNS
GAKTPLCNLIKDREPYQVCRNFLTTLMLINTNMLQISEVNQHSQSNDVSNYQINVKKENVQEYLGSSK
RFKNASFAIKDKKRKTASKGGTRNAKPAKKKPHKD

>PY01684 | Plasmodium yoelii yoelii 17XNL | hypothetical protein (Kleisin IIβ) | protein | length=773

MSTQEDEVRLLIQNLQKCNNTNECINFDLASTIQEFLNSLDKNSFEDIDKTIRENEKDKDLMNSFTSAAI
FLENCVKILGLKIEHLHNLAXNTXYNIYKENKNNNSNKKQLLIIDEEYLYINEIKNLKNTITENDIIEEDLLIK
TIPLPTFLFTDHIKVKNKIDNHKNIIKNDEKDNSLLNKKKNLNKSDIENIPYIDTLENESITSIQKNVMDLNS
EYSYQNMDNMDNMNSMENKQTLEISSVNSLNFDKLFLENDGIILLDINDYNIFINDEYDFTLQNKNSTIL
FEKYEFFSRNSIYLSNNLSEYIYEQNTIQHTYKINNIYDITSLRLCTDTLLFKTDFYSYDLALDVIKNKNYLI
NKFERQKNKLYILDETTHKNHKYNTIYKQNANYCDYCGSIIEPNESNNHCIYCNNNNEKNGFYKKLPG
YYNLSCYNITETQDFLTYMQPNKIIDIIMQNEINDTNLDANSTTNKLDQNIIDPIFHENNDSSDKSDDQKL
PNDPKLTNDQKLFKQIKIPPLYIQKLGLNIDYYYLEPLLYNLIKSLKKEKNVDRFFSINFYDHNENYDTEIL
KDDDYQEIKDEQNKTIQETLTMGTFINIKSIDNHVKNLPTSILKKTDSNTSLAFSFEDKIQDRVNKWRNF
LEKKLDILKRQPPYNVEYYKKKILKYMISNGDNIYFPDLVNDNEKYKIYRNFLTTLMLINTNKLDITEIEQN
NSNNITNYKINVKNMNVNEYINFPNSFDNIKFTINDKKRKITQNFDNKNDSLYLQKKHHI
